# Depression literacy among Australians of Chinese-speaking background in Melbourne, Australia

**DOI:** 10.1186/1471-244X-10-7

**Published:** 2010-01-19

**Authors:** Fu Keung Daniel Wong, Yuk Kit Angus Lam, Ada Poon

**Affiliations:** 1 School of Nursing and Social Work, Faculty of Medicine, Dentistry and Health Sciences, University of Melbourne, Level 5, 234 Queensberry Street, Carlton, Melbourne, Victoria, 3053, Australia; 2 Centre for Cognitive Behavioural Therapy and Training for Chinese People, Department of Social Work and Social Administration, University of Hong Kong, Hong Kong, PR China; 3 Community Settlement Services, Chinese Community Social Services Centre Inc., Melbourne, Victoria, Australia

## Abstract

**Background:**

This study investigated the knowledge of depression and preference for professional help, medications and treatment methods among Australians of Chinese-speaking background, and the perceptions of this population of the causes of mental illness.

**Methods:**

Adopting a cluster convenience sampling method, the study recruited 200 Chinese-speaking subjects from four major areas in metropolitan Melbourne where many Chinese live. The respondents were presented with a vignette describing an individual with depression and then asked questions to assess their understanding of depression and preference for professional help, medications and treatment methods. A comparative approach was used to compare the findings with those of a previous study of the mental health literacy of Australian and Japanese adults.

**Results:**

Compared to the Australian and Japanese samples, a much lower percentage of Chinese-speaking Australians (14%) could correctly identify major depression described in the vignette, and a higher percentage believed that counseling professionals could be helpful. Higher percentages of those who believed that close family members could be helpful were found in the Chinese-speaking Australian and Japanese samples, and these two groups also expressed more uncertainty about the usefulness or harmfulness of certain medications compared to the Australian sample. Higher percentages of respondents in both the Chinese-speaking Australian and the Australian sample considered "lifestyle changes" to be helpful compared to the Japanese sample. In the Chinese-speaking sample, 30%, 17.4%, 33% and 27% of the respondents rated "traditional Chinese medicine doctors," "Chinese herbal medications," "taking Chinese nutritional foods/supplements" and "*qiqong*" as helpful. Many perceived "changing *fungshui*" and "traditional Chinese worship" to be harmful. Regarding the perception of causes of mental illness, items related to psychosocial perspectives including "life stress" and "interpersonal conflict" were rated highly by the respondents, whereas traditional beliefs including "punishment for misdeeds conducted by ancestors" and "demon possession" had the lowest ratings.

**Conclusions:**

Campaigns to increase the mental health literacy of Chinese-speaking Australians are needed. The abovementioned socially and culturally driven beliefs need to be taken into consideration in the development of culturally relevant education programs.

## Background

Australia is a culturally diverse nation - 43% of Australians were born overseas or have at least one parent who was born overseas [1]. Besides English, the most commonly spoken languages in the country are Italian, Greek, Cantonese, Arabic, Vietnamese and Mandarin. The latest census shows that there are 206,590, 71,800, 24,370 and 39,970 China-born, Hong Kong-born, Taiwan-born and Singapore-born Chinese, respectively, who live in Australia [1]. People of Chinese-speaking background make up 3.4% (669,000) of the total Australian population. Overseas and Australian scholars have found that Asians, including Chinese, have a low rate of utilization of mental health services [2,3]. Underutilization is likely the result of a combination of personal, sociocultural and societal factors that influence whether or not people of culturally and linguistically diverse backgrounds seek help from mental health services. Knowledge of and beliefs surrounding mental illness and available mental health services [4,5], cultural conceptions of the causes of mental illness [6,7], public and self-stigma [8,9], the tendency to rely on informal networks for support [10], and practical difficulties in accessing services have been mentioned in the literature to be associated with delays in accessing mental health services [11].

Mental health literacy refers to "knowledge and beliefs about mental disorders which aid their recognition, management or prevention" [12]. Inherent in this concept is the assumption that individuals who have a higher level of mental health literacy will be more willing to seek professional help for themselves and/or for people whom they know may be suffering from a mental health problem. Jorm et al. conducted pioneer research [5,12] into the mental health literacy of Australians. In one of their studies [5], they found that over 67% of the 910 Australian respondents were able to correctly identify major depression described in a case vignette, and that the percentages of those who considered a family doctor/GP or counselor, antidepressants, or physical activity helpful for treating depression were 80%, 48% and 92%, respectively. In a Canadian study conducted by Wang et al. [13] using the same depression vignette, higher percentages of respondents correctly labeled the person in the vignette as suffering from depression (75.6%), considered a family doctor/GP or counselor to be helpful (89.7% and 89.3%, respectively), and said taking antidepressants or engaging in physical activities was helpful (62% and 96.5%, respectively). However, in general the mental health literacy of the Australian and Canadian samples was comparable.

Several studies have explored the mental health literacy of Chinese-speaking people. A study of the understanding of depression among Chinese-American women reported that 58% of the respondents believed that the person in the vignette suffered from a psychological disturbance, and only 13% failed to label the person's condition as depression [14]. In studies conducted by Parker and colleagues of Chinese-speaking Australians, the researchers found that (1) Chinese people tended to deny depression or express it somatically [15]; (2) Chinese were less likely to view a depressive episode as a disorder or to seek help for a psychological problem [16]; (3) most Chinese confided what they perceived to be private matters only to family members or close friends [17] and (4) many Chinese expected Western medications to provide an instant cure to all kinds of worries, without need for explanation as to how such drugs work [17]. In addition, respondents obtained knowledge about medications more often from friends and family than from medical professionals, and seldom asked their doctors questions about the drug they were prescribed because to do so would challenge their "authority" [17]. In a qualitative study of Chinese-speaking Australians conducted by Blignault, Ponzio, Rong and Eisenbruch [18], knowledge of mental illness, language barriers, stigma, confidentiality concerns, service constraints and discrimination were mentioned by the participants as major barriers affecting access to mental health services. These studies have provided us with information about the perceptions of the causes of mental illness and help-seeking behaviors of Chinese-speaking Australians. However, to the best of these authors' knowledge, there was only one study that had attempted to systematically explore the nature and level of mental health literacy among Chinese-speaking people.

This study was conducted by Klimidis, Hsiao and Minas which reported that 51% of Chinese-speaking Australians were able to recognize major depression described in a vignette, that multiple labels were used by the respondents, and that 49% and 73.6% of them said that the condition was related to emotional/mental problems and stress, respectively [19]. The authors compared their findings with those of an early study of Jorm and colleagues [4] and concluded that Chinese-speaking Australians did not appear to have a low level of mental health literacy. Their conclusion, however, warrants further validation because the two studies did not use the same vignette and response format to solicit the opinions of the two groups of respondents. In addition, the studies of Ying [14] and Klimidis [19] did not use a cultural perspective to explore the variation in the preference for the type of professional help, medication and treatment method or the conception of mental illness among Chinese-speaking people. Such an understanding would enable the design of culturally relevant public education programs to enhance the mental health literacy of people of Chinese-speaking background in Australia and other countries. In this study, we used the same depression vignette and response format proposed by Jorm et al. [20] and compared our findings with those of Jorm et al. [20] of the mental health literacy of Australian and Japanese people. We chose these two samples for comparison based on the notion that a comparison with the Australian sample might elucidate possible cultural differences in the preference for professional help, medications and treatments between Australians and Chinese-speaking Australians. On the other hand, a comparison with the Japanese sample (who are presumed to be culturally more similar to the Chinese-speaking Australians) might illuminate socioeconomic and policy differences in the preference for professional help, medications and treatments between people in these two countries. Lastly, to explore the cultural dimension of the mental health literacy of Chinese-speaking Australians, we added a number of culturally relevant options under the professional help, medication and other treatment categories in the questionnaire.

The adoption of a certain explanatory model of the causes of mental illness affects not only a person's understanding of mental illness but also his or her choice of medication and treatment [20,21]. It is therefore important to explore the cultural conception of mental illness as it is highly related to the mental health literacy of cultural groups. A study conducted by Tang et al. [22] in China found that patients who believed that they had a physical rather than a mental illness tended to seek help from qigong masters or folk healers. Studies conducted by Phillips et al. in China [6] and Wong et al. in Hong Kong [7] revealed that respondents highly endorsed psychosocial explanatory models such as stress, interpersonal conflict and personality deficits in explaining the causes of mental illness. There are, however, very few studies of the cultural conception of mental illness of Chinese-speaking Australians. Among them, Hsiao et al. [21] conducted qualitative research and found that this population combined traditional with Western medical knowledge to develop their own labels for various kinds of mental disorders, which included "mental illness," "physical illness," "normal problems of living" and "psychological problems." Parker and colleagues found that Chinese people tended to deny depression or express it somatically and did not view a depressive episode as a disorder [15-17].

## Objectives

(1) To understand the mental health literacy of Chinese-speaking Australians in Melbourne, Australia.

(2) To explore the preference for culturally specific professional help, medications and treatment methods among Chinese-speaking Australians.

(3) To understand the conception of the causes of mental illness of Chinese-speaking Australians.

## Methods

### Sample

This study adopted a cluster convenience sampling method in which subjects were taken from four major areas in metropolitan Melbourne where many Cantonese- and Putongua/Mandarin-speaking Chinese people live: Box Hill, Doncaster, Monash and Preston. The participants were recruited through social services organizations serving the Chinese population in these four areas. Posters in both traditional and simplified Chinese introducing the research were put up on the exhibition boards of these organizations. Potential participants who were interested in the study then approached the research team for further details. Those who decided to participate in the survey signed a consent form and were then given the questionnaire for completion. Selection criteria included: (1) aged 18 or above, and (2) immigrants of Chinese-speaking background who were living in Melbourne, Australia. Respondents took about 40 minutes to complete the questionnaire. The study was approved by the Ethics Committee of the University of Melbourne.

### Survey questionnaire

This instrument covered three areas: sociodemographic characteristics of the respondents, mental health literacy (depression vignette), and Chinese conception of the causes of mental illness.

#### Mental health literacy

This instrument was adapted from that used by Jorm et al. [4] to investigate mental health literacy among Australian samples. The original instrument includes depression and schizophrenia vignettes. In this study, only the vignette describing a person with major depression was used [20]. The depression vignette was written to satisfy the diagnostic criteria for major depression according to the fourth edition of the Diagnostic and Statistical Manual of Mental Disorders (DSM-IV) and tenth revision of the International Statistical Classification of Diseases and Related Health Problems (ICD-10). After reading the vignette, respondents answer a series of questions that assess their recognition of the disorder, awareness of mental illness, and beliefs about the helpfulness or harmfulness of different professions, medications and treatment methods. In the first section of this questionnaire, after the depression vignette, respondents are asked to provide written answers to two questions: "Do you think the person needs help or not?" (yes/no format) and "What do you think is wrong with the person?" The main part of the questionnaire is divided into three sections. Respondents are asked to rate each type of (1) professional, (2) medication and (3) treatment as "Helpful," "Harmful" or "Not Sure" in relation to the mental health issue faced by the person in the depression vignette. Based on a literature review [10,23,24] and our clinical experience in working with Chinese people, we added items to the list of options to explore the cultural dimension of the beliefs of respondents about professional help, medications and treatment methods. For example, under Professional Help, "traditional Chinese medicine doctor" and "traditional healer" were added; under Medications, "Chinese herbal medicine"; and under Treatment Methods, "taking Chinese nutritional foods/supplements," "qiqong," "changing fungshui" and "traditional Chinese worship." The English version of the scale was translated into Chinese and back translated into English by an experienced professional translator. Both traditional and simplified Chinese versions of the questionnaire were available for the respondents.

#### Chinese conception of the causation of mental illness

This scale measures the degree to which a respondent agrees or disagrees with different perspectives about the causation of mental illness: hereditary, biochemical, personal, environmental and cultural. It was adapted from that used in a study conducted by Wong et al. [7]. There are 23 items in the original scale, which are divided into two categories: causes of mental illness (e.g., bad fungshui), and the treatment methods associated with the causes of mental illness (e.g., rearranging the household furniture to avoid bad fungshui). However, following a review of the questionnaire conducted by a panel of three community mental health workers who had been working with Chinese people with mental illness, we decided to retain the 13 items that are related to the causes of mental illness and discard those on treatment methods because the latter items overlapped with some in the mental health literacy scale. Items on the causation scale were rated using a 5-point Likert scale ("Absolutely unrelated" (1), "Not related" (2), "Neither related nor unrelated" (3), "Related" (4), "Absolutely related" (5)). The higher is the score, the greater is the belief of the respondent that a certain perspective is related to the causation of mental illness.

## Results

Table 1 shows the demographic characteristics of the sample. There were more females than males (69.5% vs. 30.5%, respectively). The majority of the respondents were married, between 40 and 65 years of age, spoke mainly Cantonese or Putonghua/Mandarin, and were relatively well educated, having received senior secondary to tertiary education. Most of them had been born in Hong Kong or China, and migrated to Australia, on average, about 10 years previously. Fifty-six percent had an individual income of less than AUD 20000, and 18% had an income between AUD 20001 and AUD 40000; it was estimated that the weekly income of individual respondents was around AUD 450. Among them, 41% rated their English proficiency as average, and over 46% rated it as poor to very poor. About 40% of the respondents had full- or part-time jobs, and 25% were unemployed. The demographic characteristics of our sample were comparable to those of the population of Chinese-speaking Australians and immigrants described in the Australian Census [1] in terms of place of origin, major dialects spoken, length of time in Australia, average annual income and education level. However, in our sample, the male to female ratio was higher and there were more unemployed people [1].

**Table 1:** Demographic characteristics of the respondents (N = 200)

	Item	N	%
Gender	Male	61	30.5
	female	139	69.5
Age		Mean age = 49.2 Range = 19 to 78 years old	
Education	Primary school	6	3
	Secondary school	23	11.5
	Senior secondary school	57	28.5
	Diploma	48	24
	Bachelor's degree	38	19
	Master's degree or higher	12	6
	Unknown	16	8
Living in Australia (months)		Mean stay = 125 months Range = 1 to 456 months	
Place of origin	China	86	43
	Hong Kong	64	32
	Vietnam	13	6.5
	Singapore/Malaysia	18	9
	Taiwan	16	8
	Other	3	1.5
Marital status	Single	24	12
	Married	132	66
	Widow	13	3.5
	Divorced	24	11
	Cohabitating	7	7.5
Personal income per year	0-20000	112	56
	20001-40000	36	18
	40001-60000	17	8.5
	60001-80000	9	4.5
	over 80001	2	1
	Unknown	24	12
Language	Cantonese	136	68
	Putonghua/Mandarin	52	26
	Hakka	4	2
	Chiu Chou	3	1.5
	Other	5	2.5
English ability	Very poor	30	15
	Poor	63	31.5
	Average	82	41
	Good	18	9
	Very good	7	3.5
Employment	Full time	40	20
	Part time	41	20.5
	Seeking employment	37	18.5
	Unemployed	50	25
	Retired	9	4.5
	Housewife	11	5.5
	Unknown	12	6

Table 2 shows that although many respondents believed that the person in the vignette needed help, fewer of them compared to those in the other samples [20] identified the condition of the individual as major depression. They were more likely to consider the person to be suffering from stress or anxiety. The respondents in this study also rated professional help as more helpful and less harmful than did their Australian and Japanese counterparts in the study of Jorm et al. [20], and preferred counseling professionals to other professionals or lay helpers, except close family members (see Table 3). The respondents in our sample and the Japanese one both believed close family members to be helpful. Interestingly, although 30% of the respondents in our sample rated traditional Chinese medicine doctors as helpful, only 2.7% rated traditional healers as helpful, and 55% suggested the latter could be harmful (Table 3).

**Table 2:** Percentage of respondents giving labels to the depression vignette and seeing that the person needed help

Item	Australian	Japanese	Chinese Australian
Label	**%**	**%**	**%**
Depression	65.3	22.6	14 (n = 28)
Stress/anxiety	16.6	25	26.5 (n = 53)
Emotional disturbance	4.5	29.4	7.5 (n = 15)
Insomnia/lack of concentration	NA	NA	8 (n = 16)
No answer	NA	NA	44 (n = 88)
Needing help			
Yes			90.6 (n = 181)
No			9.4 (n = 19)

**Table 3:** Percentage of respondents rating each type of professional as "helpful" or "harmful" for the person in the depression vignette (by sample)

Item	%			%		
	Helpful			Harmful		
	**Australian**	**Japanese**	**Chinese Australian**	**Australian**	**Japanese**	**Chinese Australian**
1. Doctor	87.3	30.4	74	0.5	9.4	2.1
2. Pharmacist	35.4	6.8	27.6	8.7	23.6	8.6
3. Counselor	82.2	85.8	90.2	3.1	1.0	0.5
4. Social worker	62.8	73.4	86.9	4.5	1.4	1.0
5. Hot-line telephone counselor	63.5	42.4	77.4	5.9	8.6	1.1
6. Psychiatrist	65.0	69.4	73.5	7.1	5.4	3.7
7. Clinical psychologist	66.9	56.6	83.6	5.1	6.0	0
8. Close family members	67.9	85	82.2	4.9	1.6	2.1
9. Close friends	78.2	84.8	76.4	2.1	1.8	2.6
10. Naturopath	34.9	11.2	39.2	11.1	18.8	4.2
11. Religious practitioner	45.3	13.6	58.5	8.1	24.2	2.1
12. Deal with it alone	13.1	24.4	28.2	64	41.4	61.7
13. Traditional Chinese medicine doctor	NA	NA	31.8	NA	NA	8.0
14. Traditional healer	NA	NA	2.7	NA	NA	54.9

Table 4 shows that Chinese-speaking Australians were more equivocal about the helpfulness or harmfulness of certain medications on the list, especially sleeping pills, antipsychotics and tranquilizers, than the respondents in the study of Jorm et al. [20]. Higher percentages of respondents in the Chinese-speaking Australian and Japanese samples indicated that they were unsure about the usefulness or harmfulness of certain medications compared to the Australian sample. In addition, Australians generally rated medications as more harmful than did either the Chinese-speaking Australians or Japanese. Forty-one percent of Chinese-speaking Australians indicated that antidepressants would be helpful to the person in the vignette, compared to 34.8% and 46.7% of Japanese and Australians, respectively. Lastly, 17.4% of the respondents in the present study rated Chinese herbal medications as helpful. However, a similar percentage thought that they could be harmful (Table 4).

**Table 4:** Percentage of respondents rating each type of medication as "helpful" or "harmful" for the person in the depression vignette (by sample)

Item	%			%		
	Helpful			Harmful		
	**Australian**	**Japanese**	**Chinese Australian**	**Australian**	**Japanese**	**Chinese Australian**
1. Vitamins, minerals	50.2	20.2	26.8	4.4	14.6	5.7
2. St. John's wort	NA	NA	2.6	NA	NA	17.5
3. Pain relievers	14.8	4.4	6.8	37.7	43.4	55.7
4. Antidepressants	46.7	34.8	40.9	27.5	18.2	14
5. Antibiotics	10.4	6.2	37.5	38.3	29.8	4.4
6. Sleeping pills	23.9	31.6	31.1	49.6	27.0	31.1
7. Antipsychotics	11.2	22.6	27.5	48.3	19	25.9
8. Tranquilizers	13.8	38.4	26.4	60.4	15.8	29.0
9. Chinese herbal medicines	NA	NA	17.4	NA	NA	18.5

Both our subjects and the Australians in the study of Jorm et al. [20] highly endorsed the notion of lifestyle changes (i.e., engage in physical activities, get out more, learn to relax) as helpful for the person in the vignette (Table 5). However, the percentages in these areas were lower among the Japanese sample. Psychotherapy was also rated very highly by our respondents, more so than that in either the Australian or the Japanese sample. In our study, about 33% and 27% of the respondents considered consuming Chinese nutritional foods/supplements and practicing qiqong, respectively, to be helpful, whereas very few perceived changing fungshui or traditional Chinese worship to be helpful. About half of the respondents indicated that traditional Chinese worship was harmful (Table 5).

**Table 5:** Percentage of respondents rating each type of intervention as "helpful" or "harmful" for the person in the depression vignette (by sample)

	**Australian**	**Japanese**	**Chinese Australian**	**Australian**	**Japanese**	**Chinese Australian**
1. Physical activity	92.0	69.4	97.5	0.8	3.6	0.5
2. Read about problem	79.3	60	64.6	4.1	7.6	4.1
3. Get out more	87.0	67.0	60.2	0.4	3.0	8.9
4. Learn to relax	83.6	38.0	83.1	1.5	7.6	0
5. Cut out alcohol	56.0	10.0	82.6	4.7	17.2	4.6
6. Psychotherapy	44.1	49.0	89.8	10.0	7.4	0
7. Cognitive behavioral treatment	NA	NA	69.6	NA	NA	0.5
8. Hypnosis	22.4	28.0	27.8	17.0	14.2	7.2
9. Psychiatric ward	16.4	13.6	15.0	53.3	43.0	34.2
10. Electroconvulsive treatment (ECT)	5.9	2.2	9.4	69.4	50.2	16.1
11. Occasional drink	44.4	31.4	10.3	15.4	17.4	44.1
12. Taking Chinese nutritional foods/supplements	NA	NA	32.6	NA	NA	11.9
13. Qigong	NA	NA	26.9	NA	NA	9.3
14. Changing fungshui	NA	NA	5.8	NA	NA	27.7
15. Traditional Chinese worship	NA	NA	4.2	NA	NA	51.6

Table 6 documents the conceptions of the respondents in this study of the causes of mental illness. Items related to psychosocial perspective were rated highly, including life stress, negative life experience and interpersonal conflict. Personality factors including introverted personality and "think too much" also received high ratings. Traditional beliefs such as "punishment for misdeeds conducted by ancestors" and "demon possession" had the lowest ratings. The findings indicate that the Chinese-speaking Australians in this sample endorsed psychosocial rather than traditional perspectives of the causes of mental illness.

**Table 6:** Cause of mental illness as perceived by respondents

Item	Mean
1. Life stress (e.g. work, study or finance)	4.10
2. Introverted personality	3.90
3. Negative life experience	3.89
4. Think too much	3.83
5. Chemical imbalance in the brain	3.63
6. Genetic pre-disposition	3.63
7. Interpersonal conflicts	3.47
8. Too much *qiqong *practice	2.52
9. Yin Yang Imbalance	2.51
10. Fate	1.93
11. Demon possession	1.68
12. Punishment for the misdeed conducted by ancestors	1.61
13. Bad *Fung Shui*	1.58

## Discussion

### Lower percentage of depression literacy among Chinese-speaking Australians

In this study, only 14% of the respondents were able to correctly label the depression vignette, a figure that is much lower than the 67.6% for the Australian sample and slightly lower than the 22.6% observed in the Japanese sample of Jorm et al. [20]. One reason for this relatively low percentage may be related to the Chinese conception of mental illness. In recent years, there is growing evidence to suggest that Chinese people tend to view mental illness as a reflection of one's "inability to deal with social stress," "interpersonal conflict" or "personality deficits" [6,7]. Thus, it is not surprising to find that there were so few Chinese-speaking Australians in our sample who could correctly label the vignette as a case of major depression; 34% said that the person in the vignette suffered from stress/anxiety or an emotional disturbance. Indeed, our findings on the conceptions of Chinese-speaking Australians of the causes of mental illness support the abovementioned finding of other studies that Chinese-speaking Australians tend to adopt psychosocial and biochemical rather than traditional perspectives.

However, it is important to note that 44% of the respondents did not give the depression vignette any label, which might have deflated the percentage of the correct identification of the condition. It is possible that some of these respondents did know the answer, but were unsure of what label to give to the vignette and consequently chose not to give one. In the future, an answering format other than an open-ended question format may be needed to determine whether the respondent can correctly label the depression vignette.

### Endorsement of a psychosocial perspective and its influence on preferences for professional help, medications and treatment methods

In this study, Chinese-speaking Australians tended to rate counseling professionals (e.g., counselors, social workers, hot-line telephone counselors and clinical psychologists) as helpful, and the percentages of perceived helpfulness of these groups were higher than those found in either the Australian or the Japanese sample in the study of Jorm et al. [20]. Again, this may reflect the current view held by Chinese-speaking Australians that depression is caused by psychosocial problems such as stress or personality-related issues. Therefore, individuals with psychosocial problems are seen as unhappy or sad, and to require the opportunity to talk about their troubles and obtain support and assistance through counseling.

Another piece of evidence regarding psychosocial perspectives is the high rating of Chinese-speaking Australians of lifestyle changes (i.e., engage in physical activities, get out more, learn to relax) as helpful for the person in the vignette. In modern society, being active is a sign of good health. Individuals pay to participate in physical and relaxation activities such as going to the gym, doing Pilates or getting a massage to feel good and relax. These treatment methods fit the belief held by Chinese-speaking Australians that depression is a psychosocial issue and that one can overcome unhappiness through lifestyle modification.

### Endorsement of a medical perspective and its influence on preferences for professional help, medications and treatment methods

Over 70% of the Chinese-speaking Australians considered family doctors or GPs to be helpful, and this figure is comparable to that of the Australian sample. The Chinese-speaking Australians in our sample have lived in Australia for an average of more than 10 years. Thus, they may have become acculturated to Australian healthcare practice, in which a family doctor or GP is the first point of contact and gives referrals for specialized mental health services. In contrast, a fairly low percentage of respondents in the Japanese sample considered a family doctor or GP to be helpful. Jorm et al. [20] suggested, "In Japan, the interest of family doctors and GPs in psychiatric treatment is not necessarily great, and it is difficult to say that their ability to diagnose psychiatric patients correctly is sufficient." Given these findings about the perceived helpfulness of GPs, family doctors and counseling professionals, it is essential to educate GPs and family doctors in Australia about the perceptions of the causes of mental illness and preferred choice of professionals, medications and treatment methods of the Chinese-speaking population. In addition, they should be given a list of English-speaking and Chinese-speaking counseling professionals who provide services in their nearby areas so that prompt referrals can be made.

Although similar percentages of Chinese-speaking Australians and Australians rated antidepressants as helpful for the person in the depression vignette, the former were more equivocal and unsure about the helpfulness or harmfulness of certain medications, including sleeping pills, antipsychotics and tranquilizers, compared to the latter, who perceived that most of the medications were harmful rather than helpful. Such differences may reflect the difference in the level of knowledge of medications between the two groups. In Australia, GPs and pharmacists are obliged to inform their patients of the effects and side effects of medications, and Australians are generally well informed about the medications that they are taking. These patients are also more aware of their rights and more forthright in requesting information about medication from professionals. However, Chinese Confucian ethics advocate a respect for authority [22], and thus Chinese-speaking patients do not normally ask questions about the medications that they have been prescribed; rather, they simply accept what they have been given. A further barrier to knowledge about medication is that the labels and information on Western medicine are written in English and often use technical terms, making them less accessible and less easily understood by the general population of Chinese-speaking people. Although the Chinese-speaking Australians in our sample have lived in Australia for many years, the abovementioned factors contribute to their lack of knowledge about medications. Thus, it is advisable to educate Chinese-speaking Australians about the effects and side effects of medications so that they can distinguish the different types of medicines used to treat psychiatric illnesses. To help this population to better understand the medications that they are given, technical and medical terms should be avoided as much as possible and plain and ordinary language used instead.

### The influence of traditional values on preferences for professional help, medications and treatment methods

The percentage of Japanese and of Chinese-speaking Australians who endorsed close family members as helpful to the person in the depression vignette was higher than that of Australians. It is suggested that collectivism may explain the similarity between the Japanese and Chinese-speaking samples. Many studies have found that in societies with a collectivist and familial orientation, such as Japan and China, there is a tendency for people to rely heavily on their families and other close members of informal networks for support [25]. According to Wong [10], individuals in these societies do not make decisions solely on their own regarding the presence or absence of a mental illness or about seeking help from external sources; rather, they have to consider the views of different members, especially elders, before reaching a decision. Because family plays an important role in the lives of members in societies such as Japan and China, it is not surprising to find that a high percentage of first-generation Chinese-speaking Australians indicated that family members would be helpful to the person in the depression vignette.

About 30% of Chinese-speaking Australians perceived traditional Chinese medicine doctors, Chinese nutritional foods/supplements and qiqong to be helpful. Traditional Chinese medicine is widely practiced in China, Hong Kong and Taiwan, and is well respected by many Chinese. In Australia, there has been an increase in the number of traditional Chinese medicine clinics in recent years. Indeed, Chinese people do not see traditional Chinese medicine and Western medicine as incompatible [26]. Rather, they believe that Chinese medicine is useful in maintaining health and preventing illness from occurring. In China, it is not uncommon for both Western and Chinese medicine to be used to treat certain illnesses. Therefore, it is important not only to educate Chinese-speaking Australians about the advantages and disadvantages of taking Chinese herbal medicines and other treatments such as qiqong alongside Western medication but also to provide traditional Chinese medicine doctors in Australia with knowledge of psychiatric illness and mental health services so that they can be equipped to conduct initial psychiatric assessment and refer clients to GPs/family doctors so that the latter can make appropriate mental health referrals.

## Limitations

This study adopted a cluster convenience sampling method to recruit Chinese-speaking Australians in Melbourne, Australia. The research team is aware of the limitations of convenience sampling and would have preferred to use a random sampling strategy; however, this was impossible because of limited resources. To enhance the representativeness of the sample, subjects were recruited from four major areas in Melbourne where most Chinese live. However, only those who accessed the various community social services were included. Individuals who did not participate in the activities or take advantage of the services offered by these social services centers or drop by the centers would not be aware of this research project and thus are excluded from the study. Therefore, the findings might be specific to a group of relatively active Chinese-speaking participants who use the social services centers in these four areas in Melbourne, Australia. In addition, as the research was conducted in Melbourne, the results might not be generalizable to Chinese-speaking people living in other parts of Australia or overseas. Although this study used the same questionnaire and response format proposed by Jorm et al. [20], we do not know whether our sample resembles the Australian and Japanese samples of the latter study because their demographic characteristics were not given. In addition, even if the demographic characteristics are comparable, subtleties of meaning and cultural factors may influence the results of our study, and in turn, affect the interpretation of the comparison data.

## Conclusion

This study reveals the pre-existing and culturally and socially driven beliefs about mental illness, professional help, medications and treatment methods held by a group of Chinese-speaking Australians in Melbourne, Australia. Campaigns to increase the mental health literacy of Chinese-speaking Australians need to take into account these beliefs so that culturally relevant mental health education programs can be developed.

## Competing interests

The authors declare that they have no competing interests.

## Authors' contributions

FKDW initiated the research, translated and drafted the questionnaire, analyzed the data and wrote the first draft of the manuscript. YKAL contributed ideas to the design of the questionnaire and commented on the draft of the manuscript. AP and her agency helped out in the data collection. All authors have read the final manuscript and approved its contents.

## Authors' Information

Fu Keung Daniel Wong, School of Nursing and Social Work, Faculty of Medicine, Dentistry and Health Sciences, University of Melbourne, Level 5, 234 Queensberry Street, Carlton, Melbourne, Victoria, Australia, 3053. Telephone: (613) 8344-9408; fax: (613) 9347-4375; e-mail: fwong@unimelb.edu.au (Corresponding author)

Yuk Kit Angus Lam, Centre for Cognitive Behavioural Therapy and Training for Chinese People, Department of Social Work and Social Administration, University of Hong Kong, Hong Kong, China. E-mail: anguslyk@hku.hk

Ada Poon, Community Settlement Services, Chinese Community Social Services Centre Inc., Melbourne, Victoria, Australia. E-mail: ada@ccssci.com.au

## Pre-publication history

The pre-publication history for this paper can be accessed here:

http://www.biomedcentral.com/1471-244X/10/7/prepub
